# Sustained gut dysbiosis and intestinal inflammation show correlation with weight gain in person with chronic HIV infection on antiretroviral therapy

**DOI:** 10.1186/s12866-024-03431-0

**Published:** 2024-07-24

**Authors:** Aya Ishizaka, Michiko Koga, Taketoshi Mizutani, Yutaka Suzuki, Tetsuro Matano, Hiroshi Yotsuyanagi

**Affiliations:** 1 Division of Infectious Diseases, Advanced Clinical Research Center, the Institute of Medical Science, the University of Tokyo, 4-6-1 Shirokanedai, Minato-ku, Tokyo, 108-8639 Japan; 2 Department of Computational Biology and Medical Sciences, Graduate School of Frontier Sciences, the University of Tokyo, 6-2-3 Kashiwanoha, Chiba, Kashiwa-shi, 277-0882 Japan; 3 AIDS Research Center, National Institute of Infectious Diseases, Tokyo, Japan; 4 Department of AIDS Vaccine Development, The Institute of Medical Science, the University of Tokyo, Tokyo, Japan; 5 Department of Infectious Diseases and Applied Immunology, IMSUT Hospital of Institute of Medical Science, the University of Tokyo, Tokyo, Japan

**Keywords:** HIV, Microbiota, Chronic inflammation

## Abstract

**Background:**

Person with human immunodeficiency virus type-1 (PWH) are prone to chronic inflammation due to residual viral production, even with antiretroviral therapy (ART), which increases the risk of age-related diseases. There is also limited information on changes in the intestinal environment of PWH during ART. In this longitudinal study, we investigated changes in the gut microbiota, persistence of chronic inflammation, interactions between the gut environment and inflammation, and metabolic changes in PWH using long-term ART.

**Results:**

We analyzed changes in clinical parameters and gut microbiota in 46 PWH over a mean period of 4 years to understand the influence of gut dysbiosis on inflammation. Overall, changes in the gut microbiota included a decrease in some bacteria, mainly involved in short-chain fatty acid (SCFA) production, and an increase in certain opportunistic bacteria. Throughout the study period, an increase in bacterial-specific metabolic activity was observed in the intestinal environment. Continued decline in certain bacteria belonging to the Clostridia class and metabolic changes in gut bacteria involved in glucose metabolism. Additionally, patients with a low abundance of *Parabacteroides* exhibited low bacterial alpha diversity and a significant increase in body mass index (BMI) during the study period. Monocyte chemoattractant protein 1, a marker of macrophage activation in the plasma, continued to increase from baseline (first stool collection timepoint) to follow-up (second stool collection timepoint), demonstrating a mild correlation with BMI. Elevated BMI was mild to moderately correlated with elevated levels of plasma interleukin 16 and chemokine ligand 13, both of which may play a role in intestinal inflammation and bacterial translocation within the gut microbiota. The rate of BMI increase correlated with the rate of decrease in certain SCFA-producing bacteria, such as *Anaerostipes* and *Coprococcus 3*.

**Conclusion:**

Our data suggest that despite effective ART, PWH with chronic inflammation exhibit persistent dysbiosis associated with gut inflammation, resulting in a transition to an intestinal environment with metabolic consequences. Moreover, the loss of certain bacteria such as *Parabacteroides* in PWH correlates with weight gain and may contribute to the development of metabolic diseases.

**Supplementary Information:**

The online version contains supplementary material available at 10.1186/s12866-024-03431-0.

## Introduction

Although antiretroviral therapy (ART) has improved the quality of life of patients with human immunodeficiency virus (HIV) type-1 (PWH), it may also accelerate the onset of age-related comorbidities due to persistent viral production in PWH [1–3]. Continuous viral production, albeit at a low level, is a crucial factor contributing to HIV-associated immune activation and inflammation [4–7], whereas altered profiles of symbiotic gut bacteria (dysbiosis) have been suggested as host-related factors linked to inflammation [8]. Dysbiosis has been documented in numerous acute and chronic infections, including HIV [9] .

In general, HIV infection and adjacent inflammation in CD4 + T cells, which are abundant in intestinal-associated lymphoid tissues, causes disruptions to the intestinal mucosal barrier [10]. In this process, HIV-related imbalances in the gut bacteria may play a role in disease progression [11–13]. Recent studies have shown that sexual preferences affect the gut microbiota, and that the gut microbiota profile of men who have sex with men (MSM) is largely reflected in MSM HIV carriers [14, 15]. This profile may have a greater impact on the gut microbiota than the direct effects of HIV infection, and may be involved in HIV pathogenesis [13, 16–18].

Because the composition of the gut microbiota varies according to geography and sexual preferences, identifying bacteria that correlate with the pathogenesis of HIV infection is difficult in a global cross-sectional study [19]. In fact, many studies have reported changes in gut bacteria and their diversity associated with HIV infection, but there is no consensus on the exact nature of this relationship [20]. However, analyses that follow the same cohort over time are beneficial for understanding the relationship between gut microbiota changes and immunological status from post-HIV infection to treatment intervention, and beyond [21–25]. In a previous analysis of HIV-positive MSM, beta diversity of the gut microbiota differed between acutely and chronically HIV-infected and HIV-negative MSM. Changes in the gut microbiota in PWH were observed after a 12-week observation period with the ART intervention, but the differences in microbial diversity between PWH and uninfected control in diversity persisted [24]. A longitudinal analysis from naive to 2 years after ART initiation reported rapid and continuous changes in the gut microbiota and immunological recovery [22]. Microbial translocation into the blood has been reported to decrease from naive HIV patients to 48 weeks after ART [23]. However, there are some reports of immunological changes observed with the initiation of ART with no sufficient changes observed in the gut microbiota [21]. Analysis of blood markers has shown seemingly contradictory reports that the intestinal fatty acid-binding protein (I-FABP), which is released into the blood during intestinal injury, increases after ART [21, 25]. This may be due to an unknown function of I-FABP in intestinal recovery [26]. In summary, these reports showed that changes in the intestinal microbiota occur from the early stages of HIV infection and that changes in the intestinal environment proceed, to varying degrees, simultaneously with the restoration of the immune system through ART intervention.

In immunologically recovered PWH whose plasma viral load was suppressed by long-term ART, several reports, including ours, demonstrated that alpha diversity was similar to that of HIV-uninfected individuals, except that PWH had a reduced abundance of butyrate-producing bacteria, which are important for maintaining the intestinal barrier [14, 27, 28]. Our previous analysis of Japanese PWH showed that the abundance of some gut bacteria was negatively correlated with anti-inflammatory cytokines, suggesting an impact on the inflammatory environment [28]. Despite effective ART, the immune activation and inflammation associated with HIV are systemic and long-term and may involve a variety of host-side factors. In addition, co-infections, including sexually transmitted infections in PWH, also influence the composition of the gut microbiota [29–31]. This indicates that PWH (especially MSM) may be at a higher risk of changes in the gut microbiota in their daily lives than healthy individuals. In the present study, we hypothesized changes in the gut microbiota of PWH on ongoing ART and investigated their impact on chronic inflammation and metabolism. Specifically, we traced PWH, mainly MSM, from our previous study who provided stool and blood samples over the course of four years [28]. We then focused on changes in the gut microbiota during this period and performed a correlation analysis with changes in clinical parameters such as body weight and inflammatory cytokines.

## Results

### Clinical characteristics of study participants

All enrolled patients provided their first stool and blood samples between 2017 and 2018 (baseline; first stool collection timepoint). Four years after the initial gut microbiota analysis in 2018, stool and blood samples were collected and analyzed for gut microbiota in 46 PWH receiving ART treatment (follow-up study, second stool collection timepoint). Table 1 presents the demographic and clinical characteristics of the study participants. Of the 46 participants, the median age was 51 ± 12.1 years old at baseline, and 91.3% (42/46) were male. All participants had been on ART for at least 10 years, and almost all had an HIV viral load below 50 copies/mL at baseline (45/46, 97.8%). A few patients experienced periods of transient fluctuations (within 100 copies/mL) in plasma viral load; however, the viral load remained stable at less than 50 copies/mL throughout the study period. The route of HIV infection was homosexual sex in 34 cases, heterosexual sex in ten cases, injection drug use (IDU) in one case, and unknown cause in one case. The types of ART were 87% (40/46) integrase strand transfer inhibitor (INSTI)-based and 91.3% (42/46) nucleoside reverse transcriptase inhibitor (NRTI)-based, a trend consistent in the follow-up examination (Table 1). Details of individual type of ART, medical conditions, and use of luxury items (alcohol, cigarette, etc.) are listed in Table S1. Eleven (23.9%) patients did not change their ART regimen from baseline to follow-up; the remaining patients changed some medications (Table S1). The main underlying medical conditions at baseline were diabetes (*N* = 3, 6.5%), hypertension (*N* = 9, 19.6%), and hyperlipidemia (*N* = 6, 13.0%). During the study period, a few individuals were taking antibiotics, steroids, proton pump inhibitors (PPIs) or statins within 1 month before stool sample collection (Table S1). Compared with baseline data, follow-up data showed a slightly increase in body mass index (BMI), but other clinical parameters did not significantly differ (Table 1). Based on baseline BMI, 46 PWH were classified into two groups: a high (overweight, *n* = 16) and a low (normal, *n* = 30) group with a BMI cut-off of 25 kg/m^2^ for gut microbiota analysis.

**Table 1:**
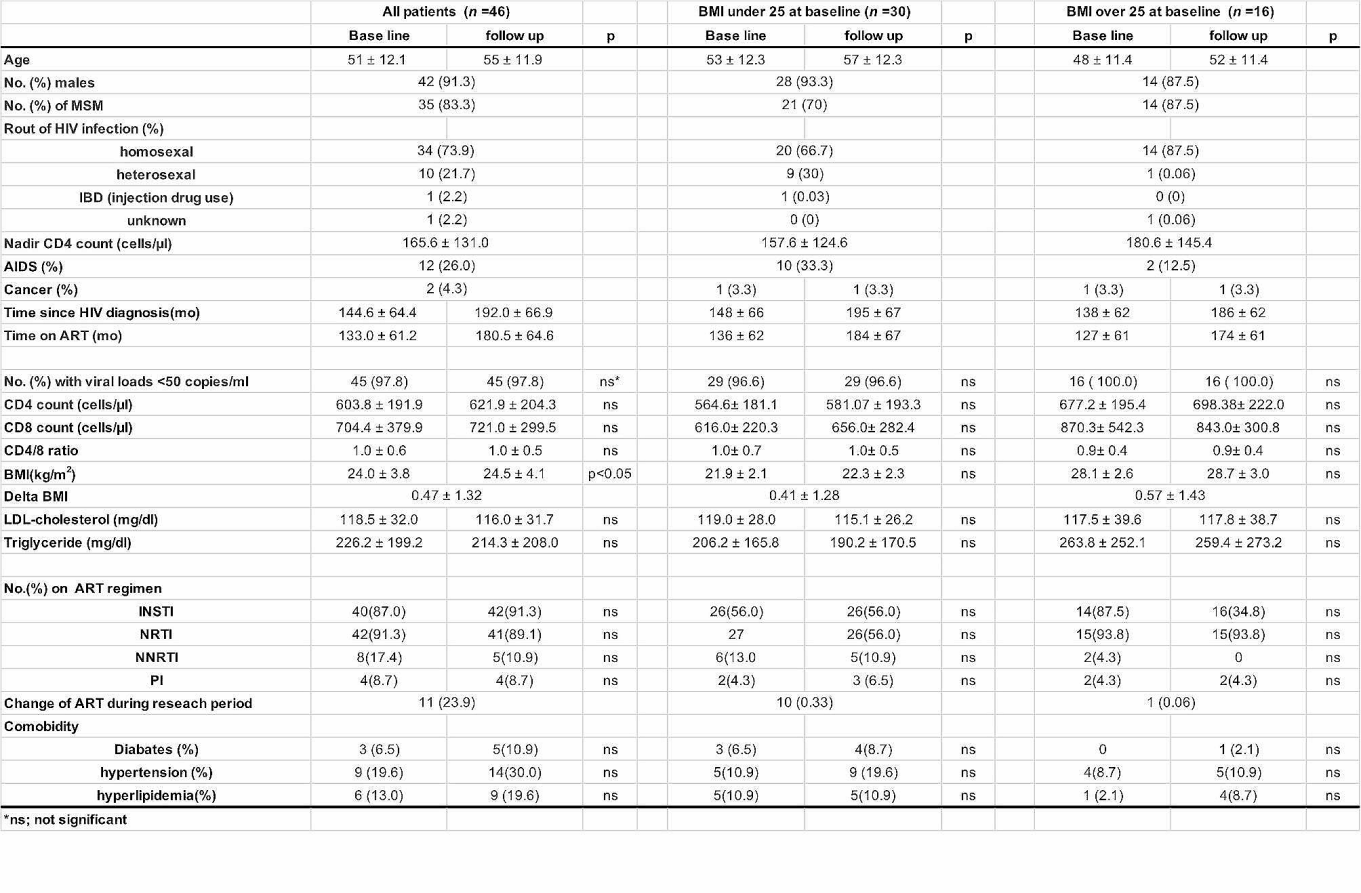
Overview of the demographic and clinical characteristics of 46 participants

### Diversity changes in the intestinal microbiota in PWH 4 years after baseline

**Fig. 1:**
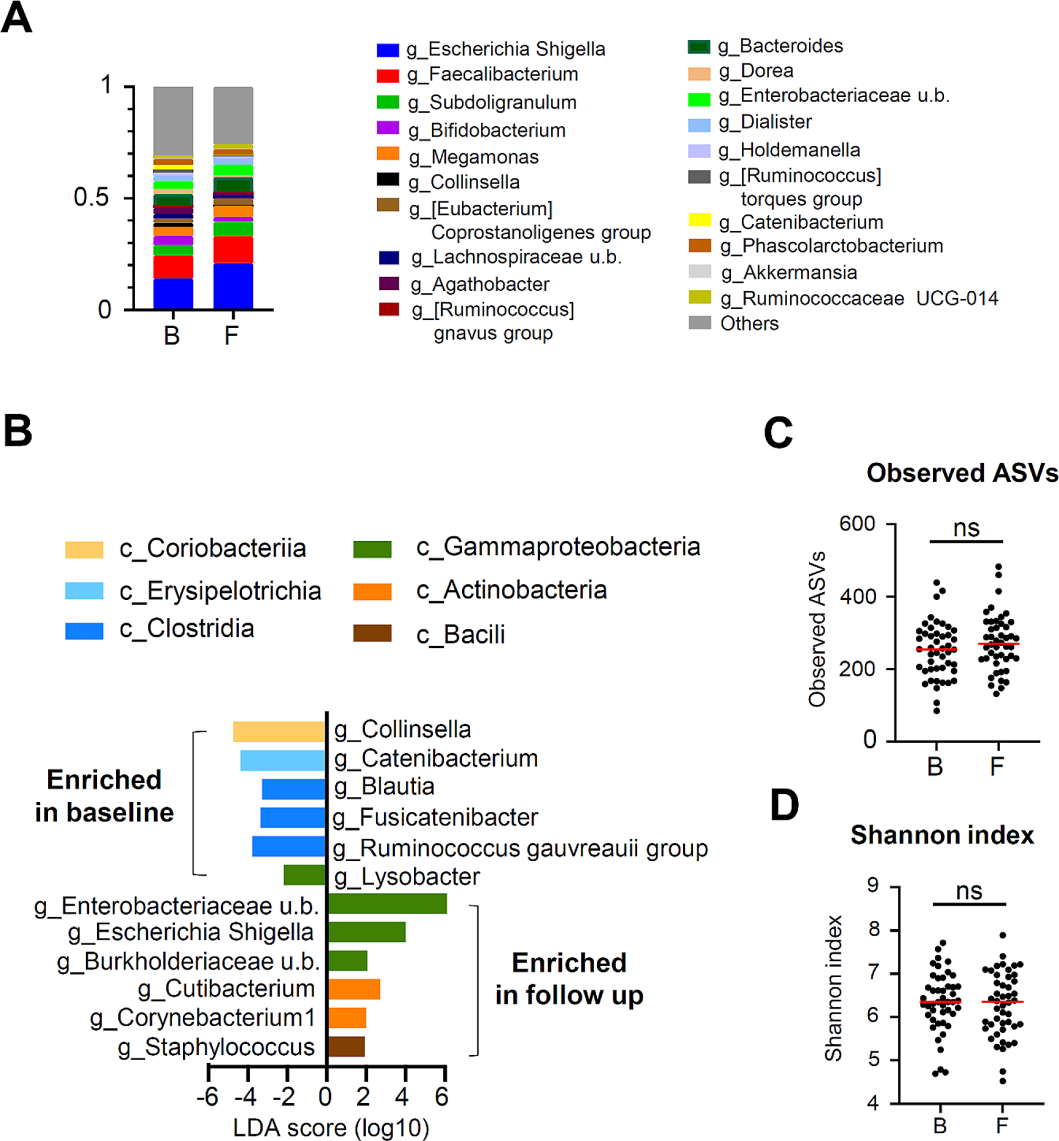
Changes in the gut microbiota and bacterial diversity analysis in people living with human immunodeficiency virus (PWH) between baseline and follow-up **(A)** Taxa bar plot of the top 20 genera of gut microbiota of 46 PWH at baseline and follow-up. **(B)** Comparative analysis of PWH gut microbiota at the genus level between baseline and follow-up based on MaAsLin2 analysis. **(C–D)** Comparative analysis of alpha diversity in PWH. Observed ASVs **(C)** and Shannon index **(D)** at baseline and follow-up

We evaluated and analyzed changes in the intestinal microbiota between baseline and follow-up. Figure 1A shows a taxa bar graph of the top 20 bacteria in terms of the presence ratio; an increasing trend in *Escherichia-Shigella* was observed in the overall bacterial microbiota movement during follow-up compared with baseline. We then analyzed the changes in the gut microbiota of PWH (Fig. 1B). During the period from baseline to follow-up, a noticeable reduction was observed in certain bacteria belonging to the Clostridia class, which are reported for their ability to produce SCFA (*Blautia*, *Fusicatenibacter*, and *Ruminococcus gauvreauii* group) [32]. Additionally, decreases were observed for *Collinsella*, *Catenibacterium*, and *Lysobacter*. In contrast, bacteria belonging to the Gammaproteobacteria class, mainly *Esherichia-Shigella*, and bacteria belonging to *Cutibacteirum*, *Corynebacterium1*, and *Staphylococcus*, were found to increase from baseline to follow-up (Fig. 1B). Moreover, we performed a comparative analysis of gut microbiota diversity between baseline and follow-up samples. In comparison to Observed amplicon sequence variants (ASVs) (Fig. 1C, upper) and Shannon index values (Fig. 1C, lower), no significant differences were observed between baseline and follow-up samples.

### Identification of gut microbiota correlated with the increased rate of BMI

**Fig. 2:**
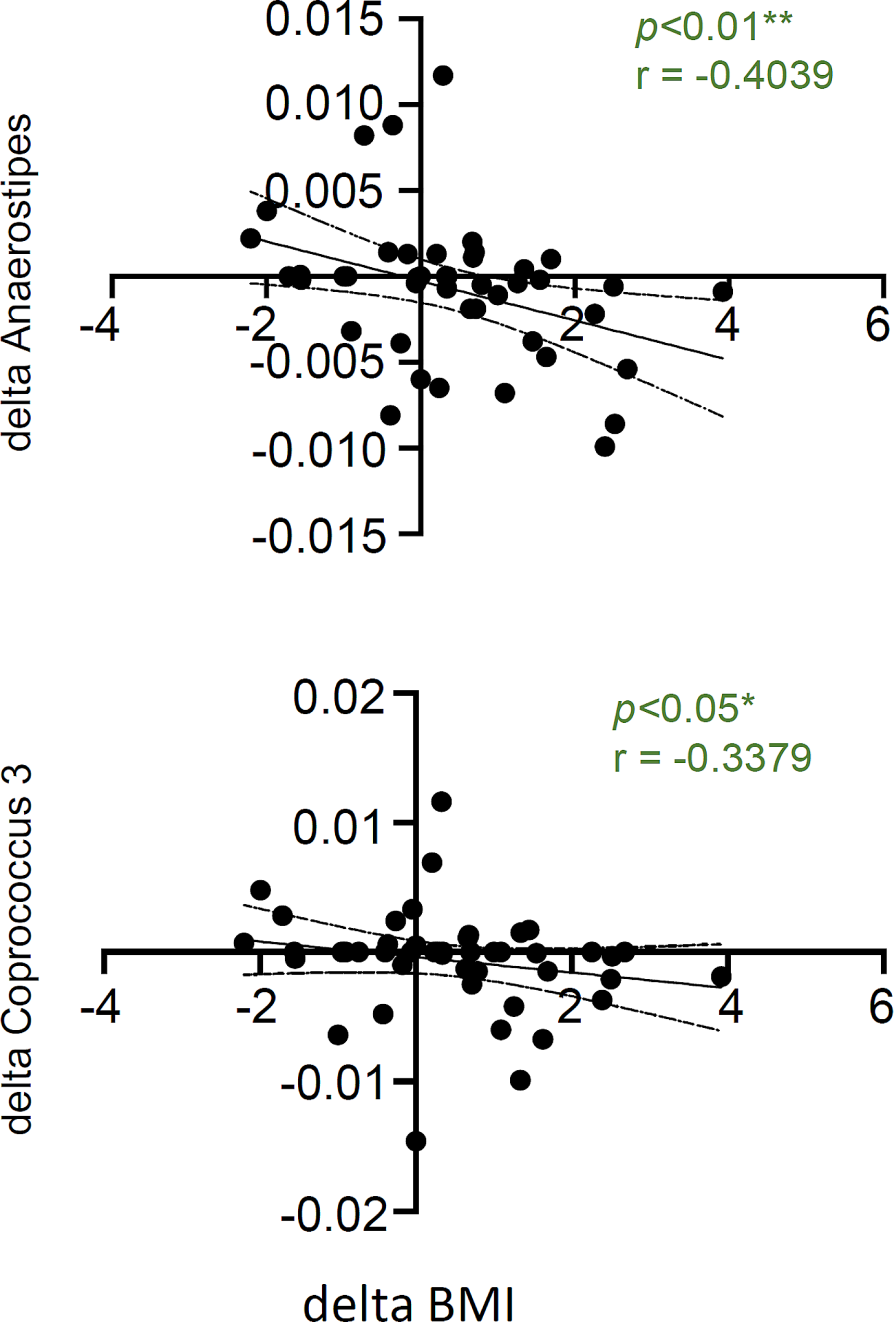
Analysis of changes in gut microbiota in the gut microbiota associated with changes in body weight **(A)** Comparative analysis based on LEfSe of the gut microbiota of the increased BMI group (BMI increased more than 1 kg/cm2 or moreover 4 years) and the remaining group **(B)** Correlation analysis of the rate of change in the abundance of gut microbiota with the rate of change in BMI from baseline to follow-up period. * *p* < 0.05, ** *p* < 0.01, u.b. unassigned bacteria

During this period, all participants’ CD4 + T-cell counts and viral loads remained unchanged (Table 1). In contrast, BMI showed an increasing trend from baseline to follow-up (*p* < 0.05), although blood cholesterol levels did not change (Table 1). Subsequently, we elucidated the relationship between the increase in BMI and bacterial microbiota. The rate of increase in BMI from baseline to follow-up (delta BMI) showed a positive correlation with the rate of decrease in *Anaerostipes* and *Coprococcus 3*, both belonging to the Clostridia Class (Fig. 2).

### Correlation between BMI and gut microbiota profile in PWH

**Fig. 3:**
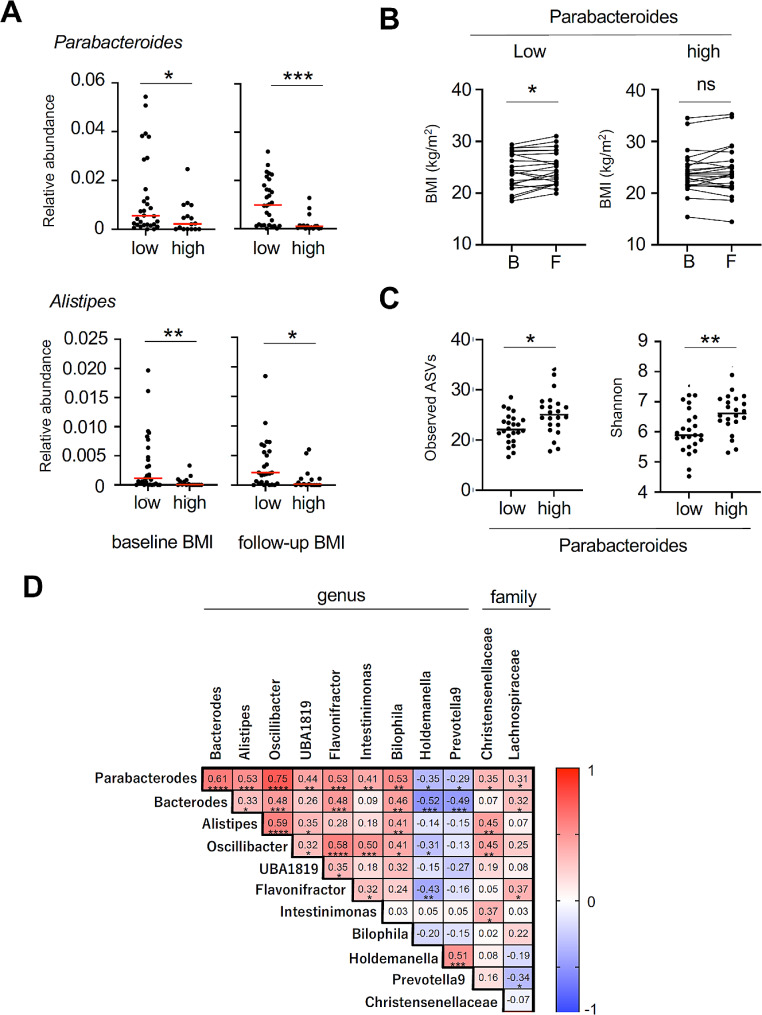
Gut microbiota associated with BMI **(A)** Comparison of Parabacteroides and Alistipes abundance at baseline and follow-up, classified into two groups based on BMI values. **(B)** Change in BMI from baseline to follow-up, classified into two groups based on the amount of *Parabacteroides* present at baseline. **(C)** Change in alpha diversity (observed ASVs and Shannon index) from baseline to follow-up, classified into two groups based on the amount of *Parabacteroides* present at baseline. **(D)** Correlation analysis with bacteria correlated with *Parabacteroides* at follow-up. * *p* < 0.05, ** *p* < 0.01, *** *p* < 0.001, **** *p* < 0.0001

Next, we attempted to identify the gut microbiota characteristic of the BMI levels of PWH. We observed that the higher BMI group exhibited a significant decrease in *Parabacteroides* and *Aristipes* from baseline to follow-up (Fig. 3A). We then divided the patients into high and low groups based on the median *Parabacteroides* abundance at baseline and observed changes in BMI. An increase in BMI was observed from baseline to follow-up in the low *Parabacteroides* group, whereas no change was observed in the high group (Fig. 3B). Additionally, the low *Parabacteroides* group exhibited a significant decrease in diversity, which was the observed ASVs and Shannon index, compared with that of the high *Parabacteroides* group (Fig. 3C). As BMI values and their changes correlated with *Parabacteroides* abundance, we extracted bacteria that correlated with *Parabacteroides* movement during the follow-up period. In addition to the genera *Bacteroides* and *Alistipes*, which belong to the phylum Bacteroidetes, the genera *Oscillibacter*, *UBA1819*, *Flavonifractor*, *Intestinimonas*, and *Bilophila*, belonging to the Clostridiales, showed a positive correlation with *Parabacteroides* (Fig. 3D). At the family level, *Christensenellaceae* and *Lachnospiraceae* were positively correlated with the abundance of *Parabacteroides* (Fig. 3D). In contrast, *Holdemanella* and *Prevotella9* showed an inverse correlation with *Parabacteroides* (Fig. 3D).

### Comparison of plasma cytokine levels and blood inflammatory biomarkers

**Fig. 4:**
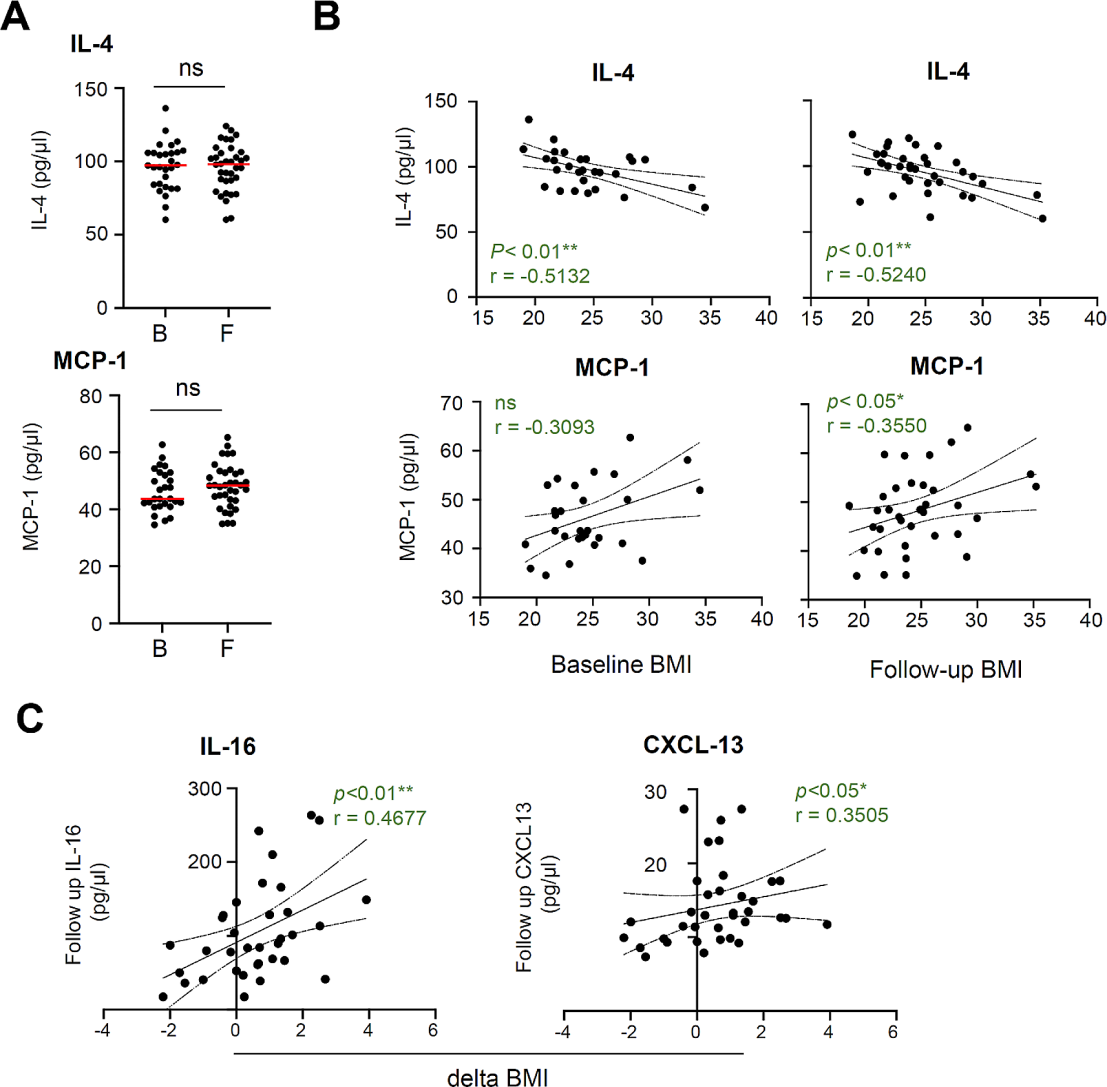
Correlation between change in plasma inflammatory biomarkers and BMI in PWH from baseline to follow-up **(A)** Comparative analysis of interleukin (IL)-4 and monocyte chemoattractant protein (MCP)-1 levels at baseline and follow-up. **(B)** Correlation analysis between BMI and IL-4 or MCP-1 at baseline and follow-up. **(C)** Correlation analysis of BMI change from baseline to follow-up with IL-16 or CXCL-13 at follow-up

BMI has been shown to correlate with chronic inflammation levels [33]. Therefore, we examined changes in plasma cytokine and chemokine levels in PWH from baseline to follow-up and extracted cytokines that correlated with BMI. Four blood markers were observed to have statistically significant correlations with BMI. Plasma interleukin (IL)-4 and monocyte chemoattractant protein-1 (MCP-1) levels did not show significant changes from baseline to follow-up (Fig. 4A); however, BMI showed positive and negative correlations with plasma IL-4 and MCP-1, respectively, at follow-up, and the correlations tended to increase from baseline to follow-up (Fig. 4B). Subsequently, we analyzed the correlation between the number of changes in BMI from baseline to follow-up and plasma protein levels at follow-up and observed that an increase in BMI positively correlated with the levels of plasma IL-16 and C-X-C motif chemokine ligand (CXCL)13 (*p* < 0.05) (Fig. 4C).

### PICRUSt2 bacterial function predictive analysis at baseline and follow-up

**Fig. 5:**
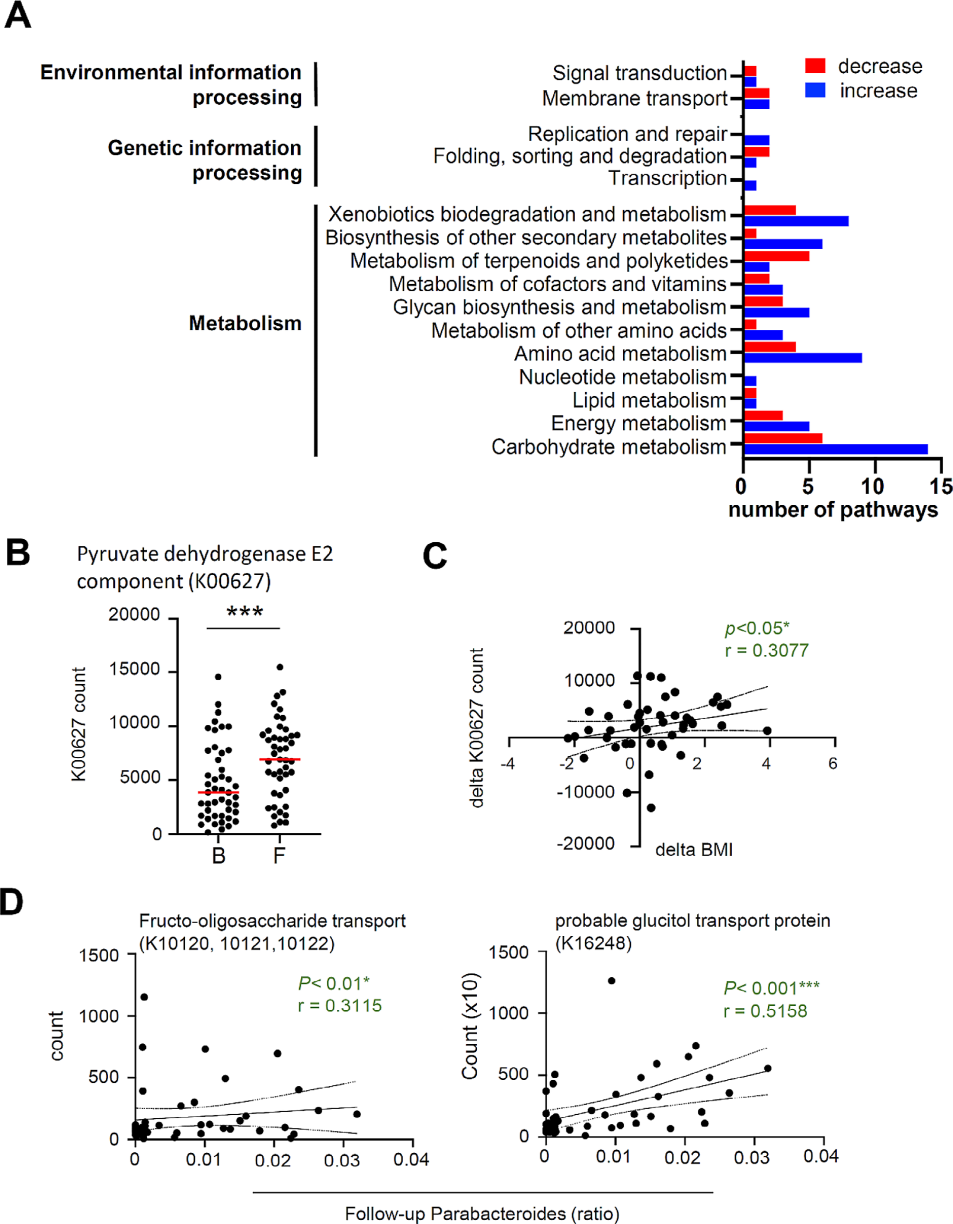
Changes in the intestinal environment of PWH predicted based on bacterial alterations **(A)** Top three functional gene pathways of Kyoto Encyclopedia of Genes and Genomes database that increased (blue) and decreased (red) at follow-up compared with baseline (q < 0.01). **(B)** Comparison of the number of functional genes for pyruvate dehydrogenase E2 component (K00627) between baseline and follow-up. **(C)** Correlation analysis of the respective percent change in BMI and pyruvate dehydrogenase E2 component between baseline and follow-up. **(D)** Correlation analysis of Parabacteroides abundance at follow-up with the estimated number of fructo-oligosaccharide transport-related genes (K10120, K10121, and K10122) (left) or the estimated number of genes for glucitol transport protein (K16248) (right) *p < 0.05, *** *p* < 0.001

We predicted the metabolic pathways with significant differences between baseline and follow-up by calculating the bacterial functional genes to understand the intestinal environment caused by changes in the bacterial microbiota from baseline to follow-up. A significant variation in 340 gene groups associated with microbial biosynthetic pathways was observed from baseline to follow-up. Figure 5A reveals the top three branches based on the biological pathways (metabolism, genetic information processing, and environmental information processing) that showed variation from baseline to follow-up. Among these variable gene groups, major changes were observed in the metabolic pathways, mainly in carbohydrate and amino acid metabolism, with a particular increase in the pyruvate dehydrogenase E2 component. This component is involved in the biosynthesis of acetyl Coenzyme A from pyruvate, a component of carbohydrate metabolism (Fig. 5B). Pyruvate dehydrogenase E2 was observed to correlate positively with the rate of increase in BMI from baseline to follow-up (Fig. 5C). Furthermore, we focused on four pathways (K10120, K10121, K10122, and K16248) that correlated with the *Parabacteroides* genus related to BMI levels (Fig. 5D). These pathways were associated with two types of bacterial microbial cell membrane transporters (K10120, K10121, and K10122), which were related to fructo-oligosaccharide transporters, and K16248, which was related to glucitol transport. These genes were positively correlated with *Parabacteroides* at follow-up, and their numbers decreased from baseline to follow-up.

## Discussion

This study observed that despite effective ART, ongoing changes in the intestinal microbiota occurred in PWH. In this follow-up study conducted 4 years later, no changes in CD4 values were observed, unlike the progressive dysbiosis of the gut microbiota. BMI increased significantly, although the difference was minimal (0.47 kg/m^2^). Furthermore, the rate of BMI increase correlated positively with increases in proinflammatory cytokines IL-16 and CXCL13 in the plasma. Increased plasma IL-16 expression in patients with ulcerative colitis is mainly limited to inflammatory areas of the colonic mucosa [34]. Plasma levels of CXCL13 correlate with leaky gut markers [35]. Experiments using human umbilical vein endothelial cells and lipopolysaccharide-induced septic mice have also shown that plasma CXCL13 plays an important role in lipopolysaccharide-induced endothelial permeability enhancement [36]. Further, one study reported that an elevated plasma levels of CXCL13 are associated with markers of microbial translocation and myeloid and lymphoid activation markers, indicating an unfavorable situation for PWH [37].

Furthermore, in this study, BMI showed positive and negative correlations with plasma levels of IL-4 and MCP-1, respectively. Animal studies have indicated that plasma IL-4, inversely correlated with BMI in our analysis, inhibits lipid accumulation in adipose tissue and is involved in weight gain and fat mass reduction [38, 39]. Plasma level of MCP-1, which has also been positively correlated with BMI [40], is secreted from white adipose tissue, induces macrophage infiltration, and contributes to obesity-induced inflammatory responses, including the dysregulation of adipocytokine production [41, 42]. Plasma MCP-1 is also associated with neurodegeneration, inflammatory bowel disease, asthma, and nephropathy [43]. One report observed that the plasma marker levels of lipopolysaccharide and MCP-1 were significantly elevated in older HIV-infected men, and these correlated with plasma sCD14, indicative of microbial translocation [44]. In other words, the increase in body weight in PWH suggests a shift to an environment with an altered quality of chronic inflammation, coupled with the addition of metabolism-related cytokines. Additionally, *Blautia*, which was observed to decrease during follow-up, has been shown to produce substances that effectively inhibit fat accumulation [45], with an inverse correlation between visceral fat area and *Blautia* levels [46]. Given these findings, our study indicates that weight gain experienced by PWH may be related to an inflammatory response associated with changes in gut microbiota diversity. Nevertheless, it is important to note that age-related weight gain has also been generally observed in individuals without HIV infection. To gain a deeper understanding of this issue, further analysis is needed, such as a comparative study of the gut microbiota in healthy individuals and PWH using animal models.

Based on the functional gene prediction using bacterial 16 S ribosomal ribonucleic acid (rRNA), a decrease in genes associated with the transport of several sugars into bacteria was observed during the follow-up study. Nondigestible and nonabsorbable carbohydrates, such as dietary fiber, are not absorbed by the body and serve as a nutrient source for intestinal bacteria [47]. In this study, decreased diversity was observed in environments with low *Parabacteroides*, negatively correlating with BMI levels. Although this study could not determine the cause of the decrease in these transporters, it may reflect the decrease in certain bacteria, mainly belonging to the Clostridia class. *Anaerostipes*, negatively correlated with increased BMI in this study, is a bacterium capable of digesting fructo-oligosaccharides [48]. Despite a reduced number of SCFA-producing bacteria, functional genes for carbohydrate and amino acid metabolic activities of bacteria were enriched during the follow-up study. These findings suggest increased bacterial metabolism activity throughout the study period. Next, the correlation between BMI and Parabacteroides observed in the present study may especially involve secondary bile acid synthesis. Recently, accumulating evidence has associated bile acid metabolism by intestinal bacteria with maintaining a healthy intestinal environment and lifestyle-related diseases related to bacterial colonization. Specifically, *Parabacteroides distasonis* and *Parabacteroides goldsteinii* have been shown to exert anti-obesity effects by producing secondary bile acids (lithocholic acid [LCA]) and ursodeoxycholic acid [49, 50]. *Parabacteroides merdae* is also involved in maintaining a healthy intestinal environment through bile acid synthesis (iso-LCA, 3-oxo-LCA, and iso-allo-LCA) [51]. Furthermore, we observed that a decrease in Parabacteroides correlated with a decrease in Christheneraceae, which has been inversely correlated with BMI [52]. Christheneraceae is often associated with dietary practices that promote health [53], particularly *C. minuta*, which is known to alter bacterial diversity, has a protective effect against obesity [54]. In the present study, a correlation with a decrease in Lachnospiraceae was observed, suggesting that the overall effect of multiple bacterial changes in PWH may contribute to metabolic changes, although no significant correlation was observed between these bacteria and inflammatory markers.

This study has some limitations. The limited number of samples analyzed requires validation using a larger sample size to generalize the conclusions. Lifestyle and pre-existing medical conditions may affect the intestinal environment independent of HIV status. In this follow-up analysis, 35 (76%) PWH had changed ART medications since the baseline time point and could not be evaluated based on the ART regimen. Additionally, we were unable to perform a comparative analysis between PWH and healthy participants because of the lack of control data at follow-up time points for comparison. Further, the study period of 4 years may not be long enough, and longer observations would be crucial to enhance the validity of the conclusions.

## Conclusions

Our data showed that despite effective ART, PWH with chronic inflammation have persistent dysbiosis, which may lead to a transition to an intestinal environment with metabolic consequences. Future research directions include understanding the role of the gut microbiota observed in this analysis in metabolic regulation, particularly secondary bile acid synthesis by the gut microbiota, and secreted products, such as bacterial metabolites and extracellular vesicles, at the molecular level.

## Materials and methods

### Participant recruitment and sample collection

Stool and blood samples were collected from 46 PWH attending the University of Tokyo Institute of Medical Science Hospital. Study participants collected baseline stool and blood samples between 2017 and 2018. Table 1 and Supplemental Table 1 present the details of participants’ ART prescriptions and pre-existing medical conditions. Blood and stool specimens were immediately transported to a laboratory near the hospital. Stool specimens were stored at -80 °C prior to deoxyribonucleic acid (DNA) preparation. Plasma fractions of blood specimens were stored at -80 °C. Clinical parameters and blood samples were obtained within 3 months before and after stool sample collection.

### DNA extraction, amplification, and 16 S rRNA gene sequencing

We extracted DNA from fecal sample-derived bacterial fractions as previously described [55]. The 16 S rRNA gene libraries were prepared following the 16 S Metagenomics Sequencing Library Preparation guide (Part #15,044,223 Rev. B; Illumina, San Diego, CA, USA). Briefly, the hypervariable V3–V4 region of the 16 S rRNA gene was amplified using specific primers: forward (5′-*ACACGACGCTCTTCCGATCT*CCTACGGGNGGCWGCAG-3′) and reverse (5′-*GACGTGTGCTCTTCCGATCT*GACTACHVGGGTATCTAATCC-3′), comprising Illumina adapter overhang nucleotide sequences (underlined). Next, adapter ligation for polymerase chain reaction amplicons was performed using NEBNext Multiplex Oligos for Illumina (Dual Index Primers Set 1; New England Biolabs, Ipswich, MA, USA). Sequencing was performed on the Illumina MiSeq system (Illumina) using the MiSeq Reagent Kit v3 (600-cycle) with a 15% PhiX (Illumina) spike-in.

### Sequencing analyses

Sequences were quality-filtered, denoised, and analyzed using Quantitative Insights Into Microbial Ecology 2 (QIIME 2 version 2021.2), as previously reported [56]. Briefly, DADA2 was used to denoise the paired end reads into ASVs [57]. A bacterial taxonomic classification was assigned to the resulting ASVs against the SILVA database (release 132) [58]. This was trimmed to the V3–V4 region of the 16 S rRNA gene using a naïve Bayesian classification method [59]. The Kruskal–Wallis test was employed for statistical analysis of alpha diversity (Shannon index) using QIIME2 software, with a cutoff value of p-value < 0.05. ASVs tables were aligned to an equal depth of 10,000 sequences per sample through alpha-rarefaction analysis to prevent any bias resulting from variations in sequencing depth.

### Statistics

Statistical analysis of metagenomic profiles was performed using multivariate analysis with linear models (MaAsLin)2 [60]. Default values of the parameters were employed, including a minimal prevalence of 0.1 and a maximum significance of 0.25, with normalization based on TSS. The R program (version 4.3.2, <https://www.r-project.org) was utilized for the MaAsLin2 analysis. Weight and aging may correlate with metabolism [61]. BMI, age, sex, antibiotic use, PPIs, statins, smoking, and alcohol consumption were considered as confounding factors in the analysis. The analysis did not involve the centered-log ratio or any other method for normalizing or transforming relative bacterial abundance data. PICRUSt2 was calculated in QUIIME2 and used to predict microbial content from sequence information in each sample and make functional predictions based on bacterial genes [62]. The Kyoto Encyclopedia of Genes and Genomes database (August 2023 version), which contains data on compounds, reactions, enzymes, and metabolic pathways that have been experimentally validated and reported in the scientific literature, was used to examine the predicted metabolic networks of gut microbiota [63]. All genus-level differential abundances of bacteria were tested using the Mann–Whitney U test. The significance of all tests was set at *p* < 0.05 or False Discovery Rate corrected *p* < 0.05 (two-tailed). GraphPad Prism 9 software (GraphPad Software, San Diego, California, USA) was used for comparative analysis. The relationship between bacterial compositions was evaluated using the Spearman correlation test in PRISM 9.

### Cytokine quantification

Inflammatory cytokine concentrations in plasma were measured using the Bio-Plex System (Bio-Rad Laboratories) with the Bio-Plex Pro human chemokine panel (40-Plex No. 171AK99MR2) and Bio-Plex Pro human inflammation panel 1 (37-Plex No. 171AL001M), following the manufacturer’s protocol. The correlation between blood markers and BMI was assessed using Spearman’s correlation coefficient in PRISM 9. The results were determined with statistically significant differences (p value < 0.05).

### Electronic supplementary material

Below is the link to the electronic supplementary material.

Supplementary Material 1

## Data Availability

Data described in this study are openly available in DNA Data Bank of Japan (DDBJ) https://ddbj.nig.ac.jp/search/en; accession number: DRA012374 and DRA017294.
